# Multifaceted characterization of the biological and transcriptomic signatures of natural killer cells derived from cord blood and placental blood

**DOI:** 10.1186/s12935-022-02697-6

**Published:** 2022-09-24

**Authors:** Haibo Gao, Min Liu, Yawei Zhang, Leisheng Zhang, Baoguo Xie

**Affiliations:** 1 Department of Endocrinology, Pingxiang People’s Hospital, Pingxiang, 337099 China; 2 Veterinary Bureau, Department Agriculture and Animal Husbandry of Inner Mongolia Autonomous Region, Hohhot, 010011 China; 3 Key Laboratory of Molecular Diagnostics and Precision Medicine for Surgical Oncology in Gansu Province & NHC Key Laboratory of Diagnosis and Therapy of Gastrointestinal Tumor, Gansu, Gansu Provincial Hospital, Lanzhou, 204 Donggangxi Road, Chengguan District, Lanzhou, 730013 China; 4 Jiangxi Research Center of Stem Cell Engineering, Jiangxi Health-Biotech Stem Cell Technology Co., Ltd, Shangrao, 334000 China; 5 Reproductive Medicine Center, the First Affiliated Hospital of Hainan Medical University, Haikou, 570102 China

**Keywords:** Natural killer (NK) cells, Perinatal blood, Cellular vitality, Transcriptome analysis, Cytotoxicity

## Abstract

**Background:**

Perinatal blood including umbilical cord blood and placental blood are splendid sources for allogeneic NK cell generation with high cytotoxicity of combating pathogenic microorganism and malignant tumor. Despite the generation of NK cells from the aforementioned perinatal blood, yet the systematical and detailed information of the biological and transcriptomic signatures of UC-NKs and P-NKs before large-scale clinical applications in disease remodeling is still largely obscure.

**Methods:**

Herein, we took advantage of the “3IL”-based strategy for high-efficient generation of NK cells from umbilical cord blood and placental blood (UC-NKs and P-NKs), respectively. On the one hand, we conducted flow cytometry (FCM) assay and coculture to evaluate the subpopulations, cellular vitality and cytotoxic activity of the aforementioned NK cells. On the other hand, with the aid of RNA-SEQ and multiple bioinformatics analyses, we further dissected the potential diversities of UC-NKs and P-NKs from the perspectives of transcriptomes.

**Results:**

On the basis of the “3IL” strategy, high-efficient NKs were generated from mononuclear cells (MNCs) in perinatal blood. P-NKs revealed comparable ex vivo expansion but preferable activation and cytotoxicity upon K562 cells over UC-NKs. Both of the two NKs showed diversity in cellular vitality and transcriptome including apoptotic cells, cell cycle, gene expression profiling and the accompanied multifaceted biological processes.

**Conclusions:**

Our data revealed the multifaceted similarities and differences of UC-NKs and P-NKs both at the cellular and molecular levels. Our findings supply new references for allogeneic NK cell-based immunotherapy in regenerative medicine and will benefit the further exploration for illuminating the underlying mechanism as well.

**Supplementary Information:**

The online version contains supplementary material available at 10.1186/s12935-022-02697-6.

## Background

Natural killer (NK) cells are advantaged innate immune cells with properties of cytolytic effector against tumor and virus infection without the need of preliminary antigen presentation, which have obtained increasing concerns since the 1970s [1–3]. Preclinical and clinical studies have indicated the potential role of alloreactive NK cells in the administration of numerous hematological malignancies and metastatic solid tumors without inducing graft-versus-host disease (GvHD) and thus resultant improved allogeneic hematopoietic stem cell (HSC)-based hematopoiesis reconstruction [4, 5]. Historically, NK cells in human are divided into the cytotoxic CD3^−^CD56^+^CD16^+^ and the IFN-γ producing CD3^−^CD56^+^CD16^−^ counterparts, which congruously and spontaneously benefit tumor immunosurveillance and elimination of cancer cells and pathogenic microorganism via natural cytotoxicity effects [1, 6]. Additionally, of the two subsets, the CD3^−^CD56^bright^ NK cells are characterized with less maturation, while the CD3^−^CD56^dim^ counterpart occupies the most numerous contents in blood [1, 7].

For decades, we and other investigators have reported the generation of NK cells from a variety of cell sources including peripheral blood, umbilical cord blood, placental blood, hematopoietic stem/progenitor cells (HSPCs) and human pluripotent stem cells (hPSCs), together with the well-established cell lines (e.g., NK-92 cell line, YT) [2, 4, 8]. To date, peripheral blood is the most utilized cell source for adoptive immunotherapy with therapeutic doses ranging from 1 × 10^6^ cells/kg to 1 × 10^7^ cells/kg [9]. Current studies have indicated the feasibility of umbilical cord blood as an alternative cell source for high-efficient NK cell generation, which are associated with advantaged properties such as faster engraftment, higher percentage of progenitors and lower risk of GvHD [10, 11]. Thus, due to the limitation of peripheral blood for large-scale NK cell generation as well as the stability of production attribute to the otherness of blood donators (e.g., age, gender, race), UCB and placental blood are recognized as the “off-the-shelf” products and readily available sources for highly functional NK cell generation and the accompanied cancer immunotherapy [1, 4, 12]. Notably, state-of-the-art updates have also reported the derivation of NK cells with enhanced cytotoxicity and in vivo persistence from human embryonic stem cells and induced pluripotent stem cells (hESCs and hiPSCs), which are promising sources for large-scale homogeneous NK cell generation, yet most of the studies sustain at the preclinical stage [11, 13]. However, the booming demands of high-quality NK cell products for immunotherapy in clinical practice are far from satisfaction, which largely due to the obscure of detailed information upon the safety and efficacy of NK cells both at the cellular and molecular levels in preclinical studies.

In the study, we initially took advantage of the splendid cytokine cocktail-based strategy for high-efficient generation of NK cells from UCB (UC-NKs)- and placental blood (P-NKs)-derived mononuclear cells (MNCs) and compared the similarities and differences both at the cellular and molecular level. In details, the UC-NKs and P-NKs showed diversity in subpopulations and cellular vitality, ex vivo activation and cytotoxicity, together with distinguishable landscape of gene expression profile and the potential biological processes as well. Collectively, our findings supply new references for systematically dissecting the biological phenotypes and transcriptome features of UC-NKs and P-NKs in future.

## Methods

### Preparation of mononuclear cells (MNCs)

Human umbilical cord blood and placental blood were obtained with the consent of the healthy donors and the approval of the Ethics Committee of Shangrao ETD-Health&Biotech Hospital and the guideline of Helsinki Declaration (KLL-2020–04). Generally, MNCs were isolated from the aforementioned umbilical cord blood (UC-MNCs) and placental blood (P-MNCs) by Ficoll-based density gradient centrifugation as we recently reported [2, 14].

### NK cell generation from MNCs

To amplify and activate NK cells in vitro, 2 × 10^6^/ml UC-MNCs or P-MNCs were cultured in NK MACS Medium (Miltenyi Biotech, Germany) supplemented with recombinant human interleukin (rhIL) addition (rhIL-2, 1000U/mL; rhIL-15, 10 ng/ml; rhIL-18, 50 ng/ml) as we recently described [2]. The culture medium was changed half the amount every 2 days for the first 7 days and then every 1 day until the 14th day. The numbers and percentages of total NK cells (CD3^−^CD56^+^) or subpopulation of NK cells from UC-MNCs (UC-NKs) or P-MNCs (P-NKs) were recorded by Trypan Blue-based viable cell counting and fluorescent antibody-based FCM assay at the indicated time points (day 0, day 7, day 10, day14), respectively. The detailed information of the cytokines was available in Additional file 1: Table S1.

### Flow cytometry (FCM) assay

FCM assay was conducted as we previously reported with several modifications [2, 15]. In details, the unstimulated MNCs (day 0) and the MNC-derived cells (day 7, day10, day 14) were harvested by centrifugation at 1200 rpm for 5 min at room temperature (RT) and washed by 1 × PBS for twice. Then, the cells were resuspended in phosphate buffer solution (PBS) contained 2% fetal bovine serum (FBS) and labelled with the fluorescence conjugated antibodies including anti-CD3-PE, anti-CD3-FITC, anti-CD4-PE, anti-CD8-PE-Cy7, anti-CD56-APC, anti-CD16-FITC, anti-NKG2D-Percp5.5, anti-NKp44-APC-Cy7, anti-NKp46-PE-Cy7, anti-NKG2A-PE, anti-CD107a-PE, 7-AAD, PI or Annexin V-FITC (BD Biosci, USA) in dark for 30 min. Finally, the cells were washed by 1 × PBS for twice and turned to FACS Canto II (BD Biosci, USA) flow cytometer for detection and FlowJo 10.0 software (Tree Star, USA) for analysis. The detailed information of the indicated antibodies was listed in Additional file 1: Table S2.

### Cell cycle analysis

Cell cycle analysis of the aforementioned MNCs and the derived cells at the indicated time points was performed as we recently described with several modifications [14, 16]. Briefly, the cells were precooled in 70% (v/v) ethanol and then fixed for 24 h at 4℃. After that, the cells were collected by centrifugation at 1000 × g for 5 min and washed with 1 × PBS at 4℃ for twice. Then, the cells were incubated with Propidium iodide (PI) staining solution for 30 min at 37 ℃ and turn to BD LSR II (BD Biosci, USA) for FCM detection and ModFit software (Verity Software House Co. Ltd, USA) for analysis.

### Cell apoptosis analysis

For apoptosis analysis, MNCs together with the derived cells were treated with the Annexin V Apoptosis Detection Kit according to the manufactures’ instructions as we described before [14, 15]. In brief, 1 × 10^6^ cells were harvested and washed with precooled 1 × PBS. After resuspension in 100 uL 1 × Binding Buffer, the cells were incubated in Annexin V-FITC for 10 min and 7-AAD solution for 5 min in dark, respectively. Finally, the percentage of apoptotic cells in MNCs or derivations was quantified with FACS Canto II (BD Biosci, USA) and FlowJo 10.0 software (Tree Star, USA).

### Cytotoxic activity assessment of NK cells

The cytotoxic activity of NK cells was conducted as we recently described [2]. For preparation, K562 cell line was cultured in RPMI-1640 basal medium (Gibco, USA) supplemented with 10% FBS (Gibco, USA) at 37 ℃, 5% CO_2_ cell incubator (ThermoFisher, USA) as we reported recently [2, 17]. The HT29 human colorectal adenocarcinoma cell line was cultured in IMDM basal medium (Gibco, USA) supplemented with 10% FBS (Gibco, USA) at 37 °C in humidified atmosphere of 5% CO2 as we previously reported [17]. For cytotoxicity assay, K562 or HT29 cells were labelled with CellTrace Violet (tested with BV421 laser channel, Invitrogen, USA) and then solely (negative control, NC) or co-cultured with NK cells derived from the indicated total mononuclear cells (MNCs) (UC-NKs, P-NKs) at a series of effector-to-target ratios (E: T). After 8 h’ coculture, the cells were collected and labeled with anti-CD3-FITC, ant-CD56-APC, anti-CD-107-PE and 7-AAD. Then the cells were washed with 1 × PBS and resuspended cells in 200 uL 1 × PBS with 5 uL Precision Count Beads (BioLegend, USA). Finally, the cells were tested by FACS Canto II (BD Biosci, USA) and data was analysed by FlowJo 7.0 software (Tree Star, USA) for detection. Cytotoxic activity assessment of NK cells was according to the formula: NK cell Cytotoxicity = (1-N_1_/N_0_) × 100%. N_1_ represents the total number of living K562 or HT29 cells in the experimental group, N_0_ represents the total number of living K562 or HT29 cells in control group [2].

### RNA-SEQ and bioinformatics analyses

For RNA-SEQ preparation, MNCs and MNC-derived NK cells (day 0, day 14) from umbilical cord blood and placental blood were harvested and lysed with TRIZol reagent (ThermoFisher, USA) according to the manufactures’ instructions as we described before [14, 16, 18]. The extracted RNAs were sent to BGI Genomics (Shenzhen, China) for sequencing. The bioinformatics analyses of the RNA-SEQ data including HeatMap, Volcano Plot, Venn diagram, Gene Set Enrichment Analysis (GSEA), Kyoto Encyclopedia of Genes and Genomes (KEGG), Gene Ontology (GO) and Principal Component Analysis (PCA) were accomplished as we recently reported [15, 16, 19].

### Statistical analysis

All data was statistically analyzed by utilizing the GraphPad Prism 8.0 software (GraphPad Software Inc., USA) as we reported before [2, 19, 20]. Student's unpaired t test was utilized for analyzing the data of two unpaired groups and one-way ANOVA was used for analyzing the data of multiple unpaired groups, respectively. Overall, we conducted experiments upon 18 samples for three independent experiments including 9 cord blood samples and 9 corresponding placental blood samples. Data was shown as mean ± SEM (N = 3 independent experiments), and statistical significance was considered only when P < 0.05. *P < 0.05; **P < 0.01; ***P < 0.001; ****P < 0.0001; NS, not significant.

## Results

### P-NKs showed preferable activation over UC-NKs but comparable proliferation

**Fig. 1:**
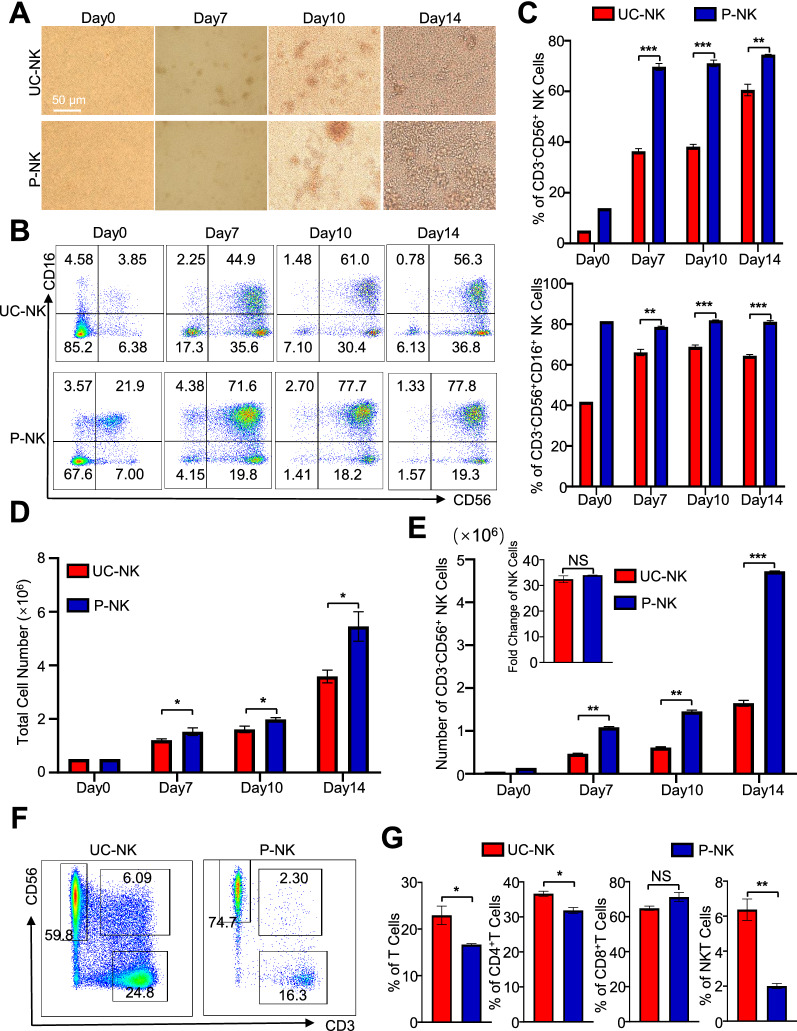
Comparison of the cytomorphology and components of UC-NKs and P-NKs. **A** Phase contrast images of UC-NKs and P-NKs at the indicated time points (day 0, 7, 10, 14) during the 14-days’ ex vivo induction. Scale bar = 50 μm. **B**, **C** FCM diagrams **B** and statistical analyses **C** of total (CD3^−^CD56^+^) and activated (CD3^−^CD56^+^CD16^+^) UC-NKs and P-NKs. **D**, **E** Statistical analyses of the numbers of total (CD3^−^CD56^+^) UC-NKs and P-NKs **D** and the activated (CD3^−^CD56^+^CD16^+^) UC-NKs and P-NKs **E**. **F**, **G** FCM diagrams **F** and statistical analyses **G** of total CD3^+^CD56^−^ T lymphocytes, CD4^+^ T cells, CD8^+^ T cells and CD3^+^CD56^+^ NKT cells in UC-NKs and P-NKs. All data were shown as mean ± SEM (N = 3 independent experiments). *, P < 0.05; **, P < 0.01; ***, P < 0.001; NS, not significant

To systematically dissect the potential similarities and differences between UC-NKs and P-NKs, we took advantage of the “3IL”-based strategy (hIL-2, hIL-15 and hIL-18) for in vitro amplification and activation of NK cells from mononuclear cells (MNCs) as we recently reported [2]. Generally, both the seeded initial UC-MNCs and P-MNCs (day 0) showed a unicellular state, followed by a clustered intermediate morphology (day 7, day 10), and finally the dense and incompact state (day 14) (Fig. 1A). As shown by the FCM diagrams and statistical analyses, a higher percentage of total CD3^−^CD56^+^ NK cells as well as the representative activated CD3^−^CD56^+^CD16^+^ NK cell subset was observed in P-MNCs (day 0) and the derivations including P-NKs (day 14) over that in the corresponding UC-MNCs and UC-NKs, respectively (Fig. 1B, C). Simultaneously, total cell number and total NK cells revealed gradual and constant increase during the process of in vitro culture, and in particular, those in the P-NKs group (Fig. 1D, E). Interestingly, the fold change of total P-NK cells was comparable to that in the UC-NKs after the 14-days’ induction (Fig. 1E). Additionally, besides NK cells, a small and higher proportion of CD3^+^ lymphocytes and CD3^+^CD4^+^ Th cells together with CD3^+^CD56^+^ NKT-like cells were observed in the UC-NKs group as well (Fig. 1F, G).

### UC-NKs and P-NKs revealed diversity in subpopulations and cellular vitality

**Fig. 2:**
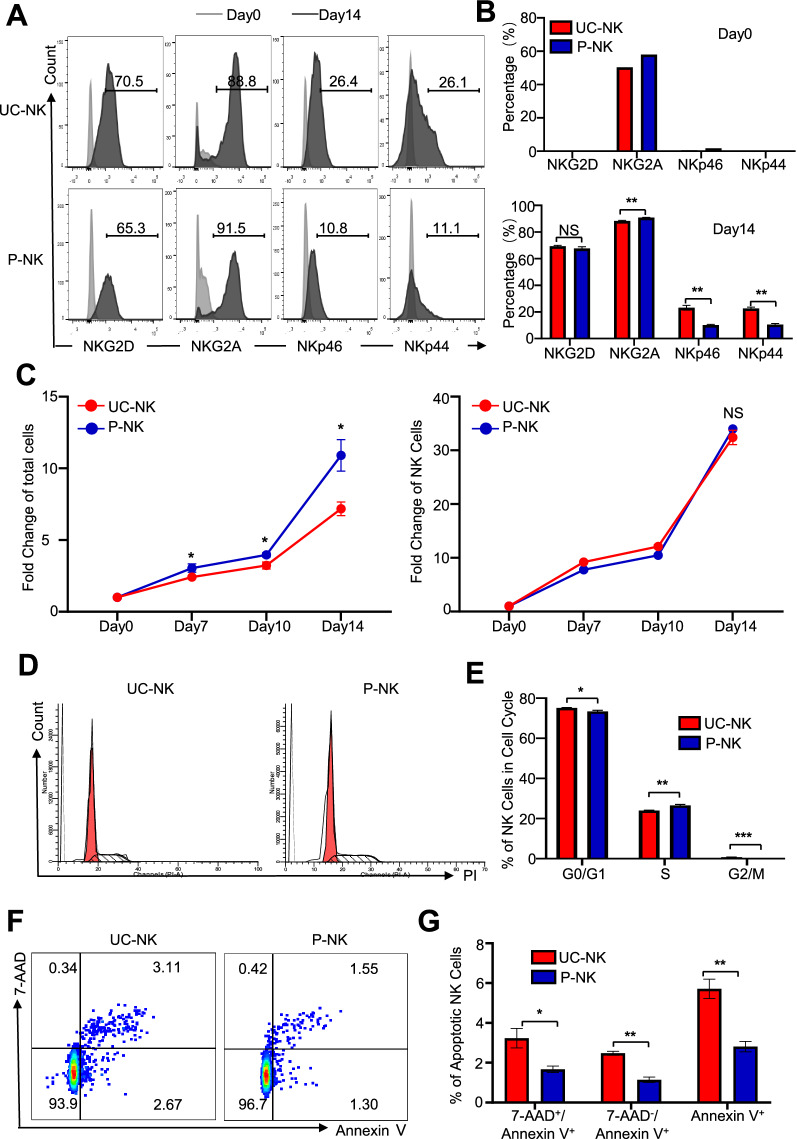
Comparison of the subpopulations and cellular vitality of UC-NKs and P-NKs **A**, **B** FCM diagrams **A** and statistical analyses **B** of the subpopulations of NK cells (NKG2D^+^, NKG2A^+^, NKp44^+^, NKp46^+^) in UC-NKs and P-NKs at day 0 and day 14 of ex vivo expansion and activation. **C** Fold change of total cells and NK cells at the indicated time points (day 0, 7, 10, 14) during the 14-days’ ex vivo induction. **D**, **E** FCM diagrams **D** and statistical analyses **E** of the distribution of sub-stages in cell cycle. **F**, **G** FCM diagrams **F** and statistical analyses **G** of the apoptotic cells in UC-NKs and P-NKs. All data were shown as mean ± SEM (N = 3 independent experiments). *, P < 0.05; **, P < 0.01; NS, not significant

In addition to generating comparable amount of NK cells from UC-MNCs and P-MNCs, we were curious about whether there’re any similarities and differences between the cytotoxic phenotype and function of the derived UC-NKs and P-NKs. For the purpose, we assessed the NK cell phenotype by monitoring the expression of various activating surface biomarkers, which were involved in regulating the dynamic equilibrium of NK cell activation and inhibition as we and other investigators reported [1, 2, 8]. As to the resting NK cells in the UC-MNCs and P-MNCs, both of them showed minimal expression of NKG2D, NKp44 and NKp46 but revealed higher level of NKG2A expression instead, which were collectively distinguish from the UC-NKs and P-NKs after ex vivo expansion (Fig. 2A, B). Interestingly, the proportion of NK cells with NKp44 or NKp46 expression in the UC-NKs group was moderately higher than that in the P-NKs group at day 14 of ex vivo culture (Fig. 2A, B).

Cellular vitality has been recognized as the fundamental for biological efficacy in cytotherapy [21, 22]. According to the curve of cumulative population, the fold change of total cells rather than the NK cells in the P-NK group was moderately higher than that in the UC-NK group (Fig. 2C). Simultaneously, the distributions of sub-stages of cell cycle in the UC-NK and P-NK groups revealed statistical differences whereas most of the cells consistently stayed in the G0/G1 stage with minimal difference (Fig. 2D, E). Furthermore, as shown by the FCM diagram based on the Annexin V and 7-AAD staining, the proportion of apoptotic NK cells in the UC-NK group after 14-days’ ex vivo expansion was a little higher than that in the P-NK group (Fig. 2F, G). Taken together, both the resting and expanded NKs (day 0, day 10) in the P-NKs group manifested diversity in cytotoxic phenotype and cellular vitality compared to the UC-NK group.

### UC-NKs and P-NKs exhibited distinguishable landscape of gene expression profile

**Fig. 3:**
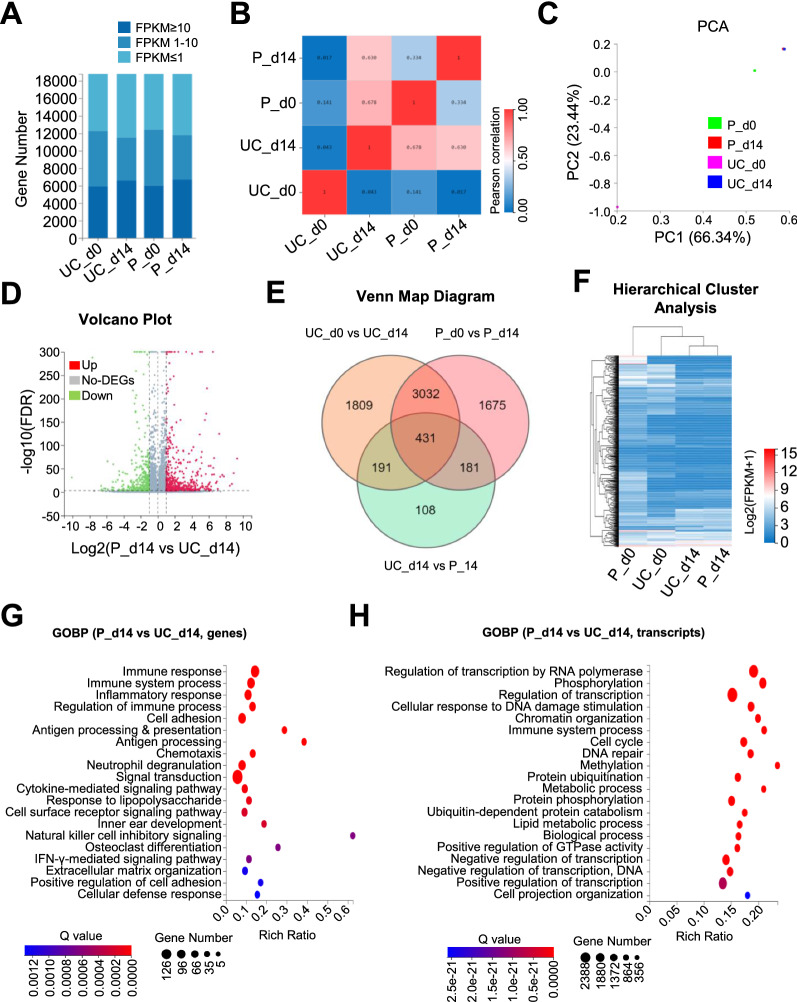
The landscape of gene expression profile of UC-NKs and P-NKs **A** The distribution of genes with various ranges of FPKM among UC-MNCs (UC_d0), UC-NKs (UC_d14), P-MNCs (P_d0), P-NKs (P_d14). **B** The correlation of the aforementioned groups. **C** Principal component analysis (PCA) of the aforementioned groups. **D** Volcano plot assay of up-regulated DEGs (Up), downregulated (Down) DEGs and non DEGs (no-DEG) between UC-NKs (UC_d14) and P-NKs (P_d14). **E**, **F** Venn map diagram **E** and Hierarchical cluster analysis **F** of DEGs among the indicated groups. **G**, **H** Gene Ontology Biological Process (GOBP) analysis of the DEGs **G** and differentially expressed transcripts **H** between UC-NKs (UC_d14) and P-NKs (P_d14)

Having disclosed the biological properties between UC-NKs and P-NKs, we next try to estimate the potentially genetic characteristics with the aid of RNA-SEQ. Generally, UC-MNCs (UC_day 0) and P-MNCs (P_day 0) as well as UC-NKs (UC_day 14) and P-NKs (P_day 14) showed more similarities in gene expression distributions, which was confirmed by the HeatMap diagram of Pearson values (Fig. 3A, B). Simultaneously, principal component analysis (PCA) of the transcriptome data including PC1 (66.34%) and PC2 (23.44%) revealed a clear clustering between UC-NKs and P-NKs (Fig. 3C).

Furthermore, volcano plot analysis intuitively exhibited the expression pattern of upregulated (Up, in red), downregulated (Down, in green) and not differentially expressed genes (no-DEGs, in gray), respectively (Fig. 3D). As shown by the Venn map diagram, a total number of 7,427 DEGs were clustered into categories seven categories (Fig. 3E). In consistence, unsupervised hierarchical clustering analysis on the basis of FPKM values of the DEGs also disclosed that UC-NKs (UC_day 14) and P-NKs (P_day 14) closer evolutionary relationship than UC-MNCs (UC_day 0) or P-MNCs (P_day 0) (Fig. 3F). To obtain further insight into the similarities and differences between UC-NKs (UC_day 14) and P-NKs (P_day 14), we conducted gene ontology (GO) analysis and found that the DEGs were principally associated with immunity (e.g., immune response, immune system process, regulation of immune process), signal regulation (e.g., cytokine-mediated signaling pathway, signal transduction, cell surface receptor signaling pathway, IFN-γ mediated signaling pathway) and antigen presentation, while the differentially expressed transcripts were mainly involved in biological process (e.g., regulation of transcription, methylation, protein phosphorylation, metabolic process) and immune system process instead (Fig. 3H).

### Potential variations of UC-NKs and P-NKs in multifaceted biological processes

**Fig. 4:**
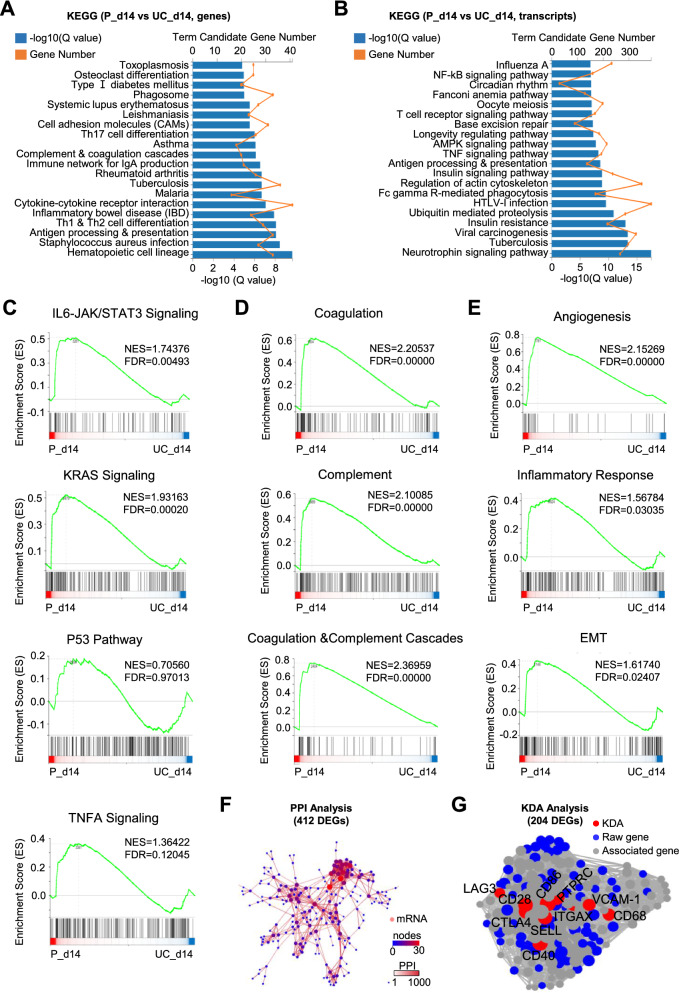
Potential variations of UC-NKs and P-NKs in multifaceted biological processes **A**, **B** KEGG analysis of the DEGs **A** and differentially expressed transcripts **B** between UC-NKs (UC_d14) and P-NKs (P_d14). **C**, **E** Potential variations of UC-NKs and P-NKs in representative signaling pathways **C** and biological processes **D**, **E**. **F** PPI analysis of the 412 DEGs (log2FC > 1.0) between UC-NKs (UC_d14) and P-NKs (P_d14). **G** KDA analysis of the 204 DEGs (log2FC > 2.0) between UC-NKs (UC_d14) and P-NKs (P_d14)

To further clarify the potential differences between UC-NKs (UC_day 14) and P-NKs (P_day 14), we took advantage of the KEGG pathway analysis, and found that cell adhesion molecule (CAM), cytokine-cytokine receptor interaction and T lymphocyte differentiation (e.g., Th1, Th2 and Th 17 cells) relevant signaling pathways were representatively enriched based on DEGs (Fig. 4A). Instead, a series of signaling pathways such as NF-kB, AMPK, TNF and neurotrophin signaling pathway were solely enriched based on differentially expressed transcripts between UC-NKs (UC_day 14) and P-NKs (P_day 14) (Fig. 4B).

In details, by utilizing the gene set enrichment analysis (GSEA), we further noticed that the datasets were uniquely enriched in immunoregulation-associated signaling pathways including IL6-JAK/STAT3 signaling (FDR = 0.00493) and KRAS signaling (FDR = 0.00020) rather than P53 pathway (FDR = 0.97013) and TNF-α signaling (FDR = 0.12045) (Fig. 4C). In consistent with the KEGG analysis, a series of tumor immunosurveillance-associated bioprocesses were representatively enriched such as coagulation, complement, coagulation and complement cascades, inflammatory response and epithelial-mesenchymal transition (EMT) (Fig. 4D, E). Furthermore, protein–protein interaction (PPI) analysis based on the STRING and DIAMOND database indicated the 412 DEGs (Log2|P-NKs/UC-NKs|> 1, Q value < 0.05, N = 412) between UC-NKs (UC_day 14) and P-NKs (P_day 14) (Fig. 4F). Additionally, with the aid of KDA analysis, the spatial distribution and interaction of the representative 204 DEGs (Log2|P-NKs/UC-NKs|> 2, Q value < 0.05, N = 204), and in particular, the pivotal immunoregulation-related genes (e.g., CTLA4, VCAM-1, CD28, ITGAX, CD68 and CD86) are intuitively displayed (Fig. 4G). Collectively, our results implicated the multifaceted variations of UC-NKs and P-NKs at transcriptome level.

### P-NKs manifested superiority in cytotoxicity over UC-NKs in vitro

**Fig. 5:**
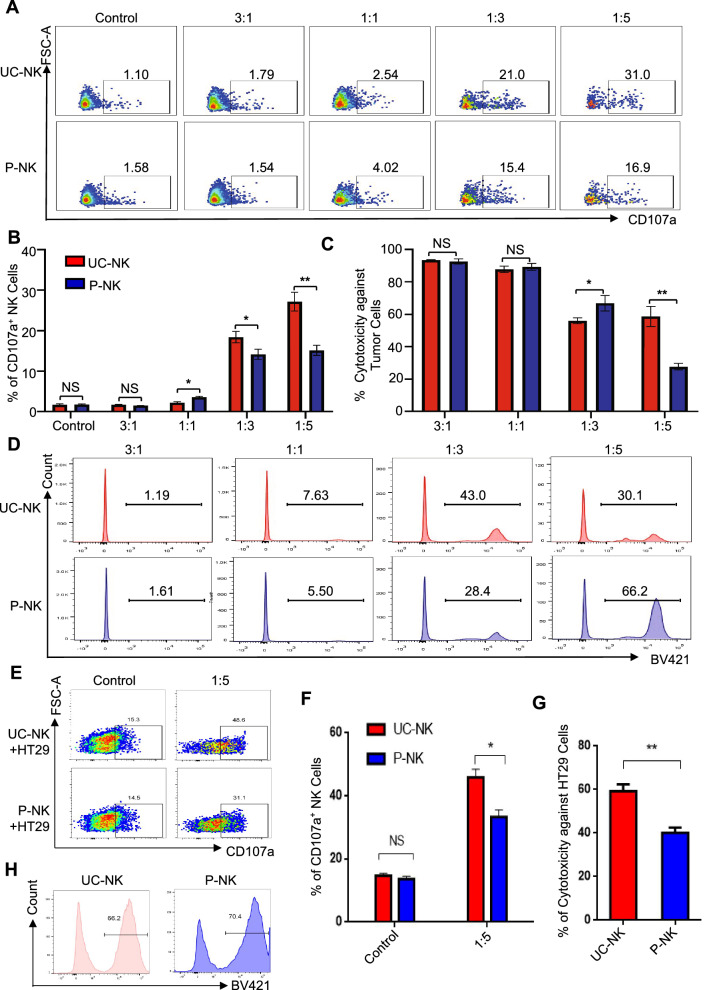
Comparison of the in vitro cytotoxicity upon tumor cell lines **A**, **B** FCM diagrams **A** and statistical analyses **B** of CD107a^+^ NK cells in coculturing with UC-NKs and P-NKs at various effector-to-target ratios (E: T = 1:1, 1:3, 1:5). **C**, **D** The comparison of the cytotoxicity of UC-NKs and P-NKs against K562 cells at the indicated E:T ratios. **E**, **F** FCM diagrams **E** and statistical analyses **F** of CD107a^+^ NK cells in coculturing with UC-NKs and P-NKs at the indicated effector-to-target ratio (E: T = 1:5). **G**, **H** The comparison of the cytotoxicity of UC-NKs and P-NKs against HT29 cells at the indicated E:T ratio (E: T = 1:5). All data were shown as mean ± SEM (N = 3 independent experiments). *, P < 0.05; **, P < 0.01; NS, not significant

Having illuminated the multidimensional diversities in cellular phenotypes and molecular features, we turn to explore the in vitro cytotoxic activity of UC-NKs and P-NKs. We took advantage of tumor-killing model by coculturing the indicated NK cells with the K562 human chronic myeloid leukemia (CML) cell line as we recently reported [2]. On the one hand, the proportions of P-NK cells with CD107a expression at most of the effector-to-target ratios (E: T = 1:1, 1:3, 1:5) was higher than those in the UC-NKs groups (Fig. 5A, B). On the other hand, P-NK cells revealed preferable killing capacity (E: T = 1:3, 1:5) over UC-NK cells according to the analysis of residual living K562 cells (Fig. 5C, D).

To further verify the cytotoxicity of the aforementioned NK cells, we conducted P-NK cells or UC-NK cell coculture with HT29 human colorectal adenocarcinoma cell line as we reported before [17]. Similarly, the percentage of CD107a^+^ P-NK cells at the indicated effector-to-target ratios (E: T = 1:5) was higher than that in the UC-NK cell group (Fig. 5E, F). Simultaneously, P-NK cells revealed preferable killing capacity (E: T = 1:5) over UC-NK cells according to the analysis of residual living HT29 cells (Fig. 5G, H). Collectively, our data indicated the moderate superiority of P-NK cells in cytotoxicity over UC-NKs in vitro.

## Discussion

State-of-the-art updates have indicated the generation of NK cells from a series of cell sources including umbilical cord blood and placental blood, yet the systematical dissection of the similarities and differences from the perspectives of biological phenotypes and transcriptome phenotypes is largely untouchable. In this study, we high-efficiently generated NK cells from UC-MNCs and P-MNCs by utilizing the “3IL”-mediated strategy as we recently reported [2, 23]. Both of the expanded UC-NKs and P-NKs exhibited typical cytomorphology and activation whereas revealed multifaceted diversities in cellular vitality, cytotoxicity and transcriptome properties.

NK cells are heterogeneous lymphocyte subpopulations originated from bone marrow HSCs and play an important role in both innate and adaptive immune response via receptor-ligand- or cytokine-mediated cytotoxicity effects and paracrine effects such as secreting perforin and granzyme, antibody-dependent cytotoxicity (ADCC) dispense with antigen presentation [1, 3, 6, 24]. In particular, the ADCC process, medicated by the therapeutic monoclonal antibodies (mAbs) and facilitated by the activating Fc receptor CD16a, has been recognized as an important effector mechanism of NK cells [23, 24]. Herein, by utilizing the “3IL”-based strategy, we efficiently derived UC-NKs and P-NKs from the corresponding MNCs with high level of CD16 expression within 14 days without gene editing. Considering the large amount of the “discarded” perinatal blood, our findings demonstrate the feasibility of exploiting umbilical cord blood and placental blood as splendid renewable sources for ex vivo expansion and activation of functionally mature NK cells with high-affinity CD16-mediated enhanced ADCC property against multiple cancers. Meanwhile, the natural cytotoxicity and cytokine production are also of great importance for mediating the functionality of NK cells. Therewith, it will be interesting to test the potential role of CD16a-associated ADCC for NK cells of both origins (UC-NK cells, P-NK cells) against a Her2/Neu breast cancer cell line or other tumor cell lines (e.g., melanoma, colon cancer cell lines) in the presence of trastuzumab or equivalent drugs in future.

For decades, numerous strategies have been developed to fulfill the clinical needs of NK cell or CAR-NK cell-based immunotherapy [8, 12, 13]. On the one hand, pioneering investigators focus on screening suitable cell sources for NK cell generation among peripheral blood (PB), NK cell lines (e.g., NK-92, NK-YS, KHYG-1), umbilical cord blood (UCB), placental blood and stem cells (HSPCs, hiPSCs, hESCs) [1, 2, 8, 11]. Generally, considering the limitation of PB-NKs and UCB-NKs in ex vivo expansion potential together with the intrinsic nature of NK-92 cells derived from Hodgkin’s lymphoma, we speculate that placenta perfusate and hPSCs hold the promising potential for large-scale clinical-grade NK cell generation [4, 25–29]. For instance, Kang et al.reported the characterization and miRNA profiling of P-NKs from placenta-derived stem cells for cancer immunotherapy, yet the systematical and detailed information of P-NKs and UC-NKs remains largely unknown [4]. Herein, we for the first time illuminated the similarities and diversities of the indicated NKs in biological phenotypes and transcriptome phenotypes. On the other hand, we and other investigators have been dedicating to develop high-efficient and cost-effective procedures for large quantities of functional NK cell generation such as feeder cell stimulation (irradiated K562 cells, K562-mbIL15-41BBL), cytokine cocktail stimulation (e.g., IL-2, IL-15, IL-18, IL-21) and even physicochemical irritation (e.g., bioreactor, culture vessels) [29–31]. In particular, NK cell expansion and functionality are largely affected by the culture medium, and thus the effects of different culture media and human serum supplementation on CB-NK or P-NK cell expansion and functionality as well as relevant cellular vitality (e.g., high dose of cytokine stimulation for activation-induced cell death) are also of great importance. For instance, Moseman and Koh reported the variations in the expansion and cytotoxicity of NK cells due to the culture medium used between the serum-free and serum-containing media formulations, respectively [32, 33]. Simultaneously, Shah et al. reported the artificial antigen presenting cell (aAPC)-mediated log-scale expansion of UC-NKs from both fresh and cryopreserved CB units with anti-myeloma activity [12]. However, mature NK cells have multiple disadvantages such as short lifespan and poor in vivo persistence [1, 34]. It’s noteworthy that Sliz and the colleagues recently reported the pivotal role of Gab3 in IL-2 and IL-15-mediated NK cell priming and expansion, together with the elimination and recognition of “missing-self” and antitumor responses, which indicated the possibility of lifespan intervention of ex vivo expanded NK cells [35]. Above all, we systematically and meticulously dissected the similarities and differences of UC-NKs and P-NKs in biological signatures, gene expression profiling and the recommended cytotoxic activity assessment for the first time. Additionally, considering the nearly 20% of total T cells remaining in the cultures at day 14, and thus all these cells would have to be removed before administration of the NK cells to the patient. Collectively, our studies would benefit the fundamental research and clinical practice of NK cell-based immunotherapy in future.

## Conclusion

In this study, we conducted multifaceted characterization of the biological signatures and transcriptomic properties of perinatal blood-derived NK cells including the UC-NKs and P-NKs, which collectively supplies new references to the preclinical and clinical application of NK cell-based cytotherapy.

## Supplementary Information

**Additional file 1: Table S1.** The cytokines used in this study. **Table S2.** Antibodies for flow cytometry assay in the study.

## Data Availability

All data of this study are included in the published article. Meanwhile, the datasets analyzed during this study are available from corresponding author upon reasonable request.

## Abbreviations

UC-NKs: Natural killer cells from umbilical cord blood
P-NKs: Natural killer cells from placental blood
FCM: Flow cytometry
MNCs: Mononuclear cells
GvHD: Graft-versus-host disease
HSC: Hematopoietic stem cell
hPSCs: Human pluripotent stem cells
HSPCs: Hematopoietic stem/progenitor cells
PBS: Phosphate buffer solution
FBS: Fetal bovine serum
GSEA: Gene set enrichment analysis
EMT: Epithelial-mesenchymal transition
PPI: Protein-protein interaction
CML: Chronic myeloid leukemia
ADCC: Antibody-dependent cytotoxicity
mAbs: Monoclonal antibodies
PB: Peripheral blood
aAPC: Artificial antigen presenting cell
