# Supplementary material for: Multifaceted characterization of the biological and transcriptomic signatures of natural killer cells derived from cord blood and placental blood
Source: Cancer Cell Int. 2022 Sep 24;22:291. doi: 10.1186/s12935-022-02697-6 (PMC9508758; doi:10.1186/s12935-022-02697-6)
Supplement: Supplementary file 1 — Additional file 1: Table S1. The cytokines used in this study. Table S2. Antibodies for flow cytometry assay in the study. [file 12935_2022_2697_MOESM1_ESM.docx]

**Supplementary Information**

**Multifaceted Characterization of the Biological and Transcriptomic Signatures of Natural Killer Cells Derived from Cord Blood and Placental Blood**

Haibo Gao^1^**^§^**, Min Liu^2^**^§^**, Yawei Zhang^1^, Leisheng Zhang^3,4*^, Baoguo Xie^5*^

**Supplementary Information**

**Supplementary Table S1-S2**

**Supplementary Table S1. The cytokines used in this study.**

| **Reagent** | **Cat. No.** | **Conc.** | **Source** |
| --- | --- | --- | --- |
| Recombinant Human IL-2 (rhIL-2) | 200-02 | 100 ng/uL | PeproTech Inc, USA |
| Recombinant Human IL-15 (rhIL-15) | 200-15 | 10 ng/uL | PeproTech Inc, USA |
| Recombinant Human IL-18 (rhIL-18) | 119-BP-100 | 10 ng/uL | R&D Systems, USA |

**Supplementary Table S2. Antibodies for flow cytometry assay in the study.**

| **Antibody** | **Cat. No.** | **Source** |
| --- | --- | --- |
| Anti-CD3-PE | 981004 | BioLegend |
| Anti-CD3-APC-Cy7 | 300316 | BioLegend |
| Anti-CD4-PE | 357403 | BioLegend |
| Anti-CD8-PE-Cy7 | 344711 | BioLegend |
| Anti-CD16-FITC | 302005 | BioLegend |
| Anti-NKG2D- Percp-cy5.5 | 320817 | BioLegend |
| Anti-CD56-APC | 362503 | BioLegend |
| Anti-CD56- Percp-cy5.5 | 362505 | BioLegend |
| Anti-CD107a- PE-Cy7 | 328617 | BioLegend |
| 7-AAD-Percp-cy5.5 | 559925 | BD Pharmigen |
| PE anti-human IgG | 409304 | BioLegend |
| Percision Count Beads | 424902 | BioLegend |
| DAPI | MBD0015 | Sigma-Aldrich |
| Cell Cycle and Apoptosis Detection Kit | C1052 | Beyotime Biotehnology |
| CellTrace Voilet | C34557 | Invitrogen™ |
| Annexin V-FITC | AO2001-02G | Tianjin Sungene Biotech |
| Annexin V binding buffer (10X) | AB2000-G | Tianjin Sungene Biotech |
